# Effects of Maitland mobilization technique on upper extremity function in stroke survivors with spasticity: An experimental study

**DOI:** 10.1097/MD.0000000000038184

**Published:** 2024-05-17

**Authors:** Ziqing Liu, Zhangjie Li, Chaoyang Duan

**Affiliations:** a Department of Rehabilitation, Shanghai Yangpu Hospital of TCM, Shanghai, China; b Department of Rehabilitation, Yangpu Hospital, Tongji University, Shanghai, China.

**Keywords:** Maitland joint mobilization, muscle tone, spasticity, stroke

## Abstract

**Background:**

The recovery of upper limb function is of great significance for stroke patients to regain their self-care ability, yet it is still a difficult point in clinical practice of neurological rehabilitation. This study aimed to investigate the effect of Maitland joint mobilization technique on the recovery of upper extremity function in patients with spasticity after stroke.

**Methods:**

From August to December 2023, 71 patients with upper extremity flexor spasm after stroke were recruited and randomly divided into experimental group (n = 35) and control group (n = 36). The control group was given conventional rehabilitation treatment, while the experimental group was treated with Maitland mobilization technique treatment of upper extremity joints on the basis of the control group. The experiment lasted for 8 weeks. Participants of the 2 groups were observed for Fugl-Meyer motor assessment-upper extremity (FMA-UE), box and block test (BBT) and Brunnstrom stage, modified Ashworth scale (MAS), and functional independence measure (FIM) at pre- and post-8 weeks study.

**Results:**

There was no significant difference in gender distribution, hemiplegic side, diagnosis, past history, age, duration, body mass index, and mini-mental state examination between the 2 groups (*P* > .05). After 8 weeks of intervention, both groups showed significant improvement in FMA-UE, Brunnstrom stage, BBT, FIM, and MAS of the shoulder (*P* < .05); however, there was no significant change in MAS of the elbow, wrist, and finger joints (*P* > .05). The posttreatment values showed a significant improvement in FMA-UE, BBT, and FIM in the experimental group compared to the control group. Comparing the changes in pretreatment and posttreatment, FMA-UE, BBT, and FIM in the experimental group were significantly improved compared with those in the control group (*P* < .05).

**Conclusion:**

Maitland joint mobilization can improve the motor function of upper extremity and the spasticity of shoulder joint complex in patients with stroke.

## 1. Introduction

Stroke is one of the most important cardiovascular diseases that threaten human-being society today, with the characteristics of high morbidity, high mortality, high recurrence rate, and high disability rate. According to the World Health Organization (WHO) statistics, in recent years, nearly 20 million people died of cardiovascular and cerebrovascular diseases every year in the world, and the mortality rate of diseases including stroke is increasing year by year.^[1]^ Stroke is also the second most common cause of death worldwide after acute myocardial infarction.^[2]^

Stroke survivors with hemiplegia usually have different levels of motor dysfunction in the recovery period, which is the main cause leading to disability in stroke patients. The problem of dystonia is the key and difficult point in the functional recovery after stroke. Limb spasticity caused by excessive muscle tension is an important obstacle for stroke patients to resume self-care and return to normal work and daily life. Spasticity of the hemiplegic side of the upper extremity (usually upper extremity flexor spasticity) directly affects the ability of daily living and quality of life of patients.^[3]^ Spasticity, on the other hand, can also lead to a higher medical burden.^[4]^ At present, the clinical physical therapy of upper extremity spasticity in stroke includes acupuncture in traditional Chinese medicine,^[5]^ neurodevelopmental therapies such as Bobath, Rood, and PNF techniques in physical medicine and rehabilitation field, such as extracorporeal shock wave,^[6]^ neuromuscular electrical stimulation^[7]^ and nerve regulation technology.^[8,9]^ For patients with severe spasticity, muscle relaxants such as baclofen can also be taken orally, while clinicians should pay close attention to the dosage, timing and adverse reactions of drug intervention^[10]^; at the same time, there are surgical approaches such as nerve block and transplantation.^[10,11]^ Among them, neurodevelopmental therapy is the main physical therapy approach for spasticity intervention in rehabilitation medicine,^[12]^ however, there is a problem of low treatment efficiency.

Maitland joint mobilization technique is one of the mainstream physical therapies in the field of musculoskeletal rehabilitation, which is mainly used to manipulate with bone and joint pain and limited movement caused by various reasons, such as functional impairment with chronic neck pain^[13]^ and temporomandibular joint dysfunction.^[14]^ Although there are few reports on the application of this physiotherapy technique in neurological rehabilitation, the clinical experience in the field of musculoskeletal rehabilitation suggests that joint mobilization technique can reduce the muscle tension of extremity, enhance the proprioception of joints, and help to establish normal movement patterns.^[12,15]^ Due to the long-term spasticity of the affected extremity after stroke, it will inevitably lead to the physiological structure disorder and decreased proprioception of the bones, joints, and soft tissues of the limbs. Therefore, this study attempted to apply Maitland joint mobilization technique to the rehabilitation treatment of upper extremity in stroke patients, and conducted a prospective randomized controlled clinical trial to explore the application value of Maitland technique in spasticity intervention, so as to optimize the existing neurological rehabilitation treatment programme.

## 2. Methods

### 2.1. Study design

This study is a prospective, single-center, single-blind randomized controlled clinical trial from August to December 2023 conducted in the rehabilitation medical center of our hospital. Stroke patients with upper extremity flexor spasm were recruited and randomly divided into experimental group and control group. The control group received routine physical therapy, while the experimental group performed Maitland mobilization technique therapy for each joint of the hemiplegic upper extremity on the basis of the control group. A total of 8 weeks of intervention were conducted in the experiment, and rehabilitation indicators were evaluated for both groups of patients before and after the experiment. All patients were evaluated by a Physiotherapist who had received professional training and did not know about grouping and intervention. All patients or their guardians are aware of the experimental intention and participation benefits, and voluntarily sign a written informed consent form. This experiment strictly abided by the principles of the Declaration of Helsinki, relevant laws and regulations, and clinical operation specifications, and was approved by the Ethics Committee of Yangpu TCM Hospital (Approval No. SHYP-2023-LC-01).

### 2.2. Sample size

This study is a randomized controlled trial, the experimental group is the joint mobilization treatment group, the control group is the conventional rehabilitation treatment group. The effective rate of treatment is used as the observation outcome index to estimate the sample size. According to the previous small sample clinical trials, the effective rate of the experimental group was 85%, and the effective rate of the control group was 52%. Assuming that the bilateral *α* = 0.05, the grasp degree was 80%. According to the sample size calculation formula “*n* = 2 $\bar{p}\bar{q}$ (*z*_***α***_ + *z*_***β***_)^2^/(*p*_1 −_ *p*_2_)^2^,” the sample size was 32 cases in each group and 64 cases in the 2 groups. Taking into account the loss of follow-up and refusal of 15%, it was concluded that at least 74 subjects should be included in the 2 groups.

### 2.3. Participants

Seventy-four stroke spasticity patients hospitalized in the Department of Rehabilitation Medicine and Neurology of xxx Hospital from August to December 2023 were recruited. Inclusion criteria: diagnosed by CT or MRI, aged 18 to 80, duration ≥ 3 months, in stable condition, manifested as spastic paralysis of one limb (mainly upper extremity flexor spasm). Flexion/extension manual muscles testing of elbow and wrist > 2/5, modified Ashworth score (MAS) < 3/5 for the same muscle groups, the Brunstrom stage of the hemiplegic upper extremity was stage II or above, no severe pain and obvious limitation of joint movement in the shoulder joint complex, elbow joint, wrist joint, and interphalangeal joint of the upper extremity on the hemiplegic side; be able to understand and carry out the instructions of the physical therapist and family members, and the patient or his/her guardian can sign the informed consent voluntarily. Exclusion criteria: patients with massive cerebral infarction or cerebral hemorrhage, aged over 80, and in unstable condition; patients with severe heart disease, bleeding dysfunction, hypertension, hyperglycemia that could not be effectively controlled, or undergoing treatment for malignant tumors; patients with mental dysfunction who could not cooperate with rehabilitation training. Withdraw criteria: those who did not meet the inclusion criteria of this experiment and entered by mistake; who failed to complete the treatment according to the experimental regulations for any reason.

### 2.4. Randomization

Subjects who met the requirements of the study were randomized in an equal ratio (1:1). Using the method of random number function “= RANDBETWEEN (1,100),” 74 random integer numbers from 1 to 100 were generated in the Office 2013 version of Excel software, and the random numbers were sorted in ascending order and then grouped according to the same proportion, that is, the first 37 numbers were set as the experimental group. The last 37 were set as the control group. The group data of the subjects were encrypted and kept by a physical therapist who did not participate in the experiment.

### 2.5. Interventions

Participants in both groups received routine rehabilitation treatment for stroke. On the basis of receiving routine rehabilitation treatment, the experimental group also received Maitland mobilization additionally of hemiplegic upper limb joints. Neither the physical therapist nor the occupational therapist who administered the intervention was aware of the patient’s grouping.

#### 2.5.1. Rehabilitation therapy

Routine rehabilitation treatment is mainly to improve the range of motion of upper extremity joints, enhance muscle strength, endurance, coordination, and the fine function training of hands. Physical therapies performed neurodevelopmental therapy (Bobath concept, PNF, and Rood technique), upper extremity free hand stretching training, passive movement training of upper extremity joints, task-oriented training and constraint-induced movement therapy, once a day, 45 min each time, 5 times a week, totaling 8 weeks. Finger grasping flexion and extension training, MOTOmed upper extremity training, roller, frosted board, picking beans and screwing, once a day, 30 minutes each time, 5 times a week, a total of 8 weeks.

#### 2.5.2. Maitland joint mobilization

**Figure 1.**
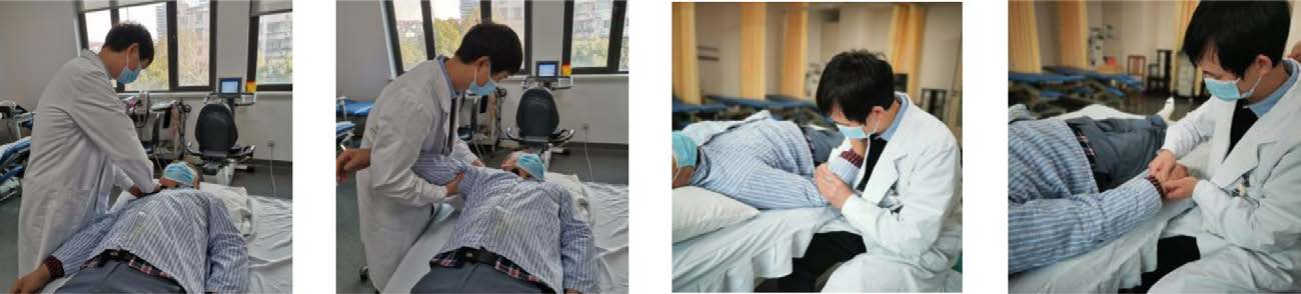
The figure shows the Maitland mobilization, including soft tissue release of the upper thoracic opening of the hemiplegic upper extremity, shoulder joint complex (acromioclavicular joint, sternoclavicular joint, scapulothoracic joint, and glenohumeral joint), elbow joint, wrist joint, metacarpophalangeal joint, and interphalangeal joint. Maitland mobilization technique refers from *Maitland’s peripheral manipulation (2005*).^[15]^

The Maitland joint mobilization procedures in the experimental group were performed according to the *Maitland’s peripheral manipulation (2005*),^[15]^ including soft tissue release of the upper thoracic opening of the hemiplegic upper extremity, shoulder joint complex (acromioclavicular joint, sternoclavicular joint, scapulothoracic joint, and glenohumeral joint), elbow joint, wrist joint, metacarpophalangeal joint, and interphalangeal joint (Fig. 1). The patients were treated with passive mobilization once a day, 30 minutes each time, 5 times a week for 8 weeks.

### 2.6. Assessments

All participants were evaluated before and after treatment in terms of nerve function recovery stage, muscle tension, upper extremity motor function and self-care ability. Brunnstrom Stage was used in the stage of nerve function recovery, and MAS was used to assess muscle tension. Fugl-Meyer motor assessment-upper extremity (FMA-UE), and box and block test (BBT) were used for upper extremity motor function. The functional independence measure (FIM) was used to evaluate the self-care ability. All outcomes were assessed by the same physiotherapist, who was not involved in the therapeutic intervention and grouping of patients.

#### 2.6.1. Fugl-Meyer motor assessment-upper extremity

The scale is mainly used to evaluate the recovery of upper extremity motor function in stroke patients with hemiplegia. The scale can be divided into 33 items, each item can be divided into 0, 1, and 2 points according to the completion of the patient’s action, a total of 0 to 66 points. The higher the score, the better the recovery of upper extremity motor function.^[16]^

#### 2.6.2. Box and block test

**Figure 2.**
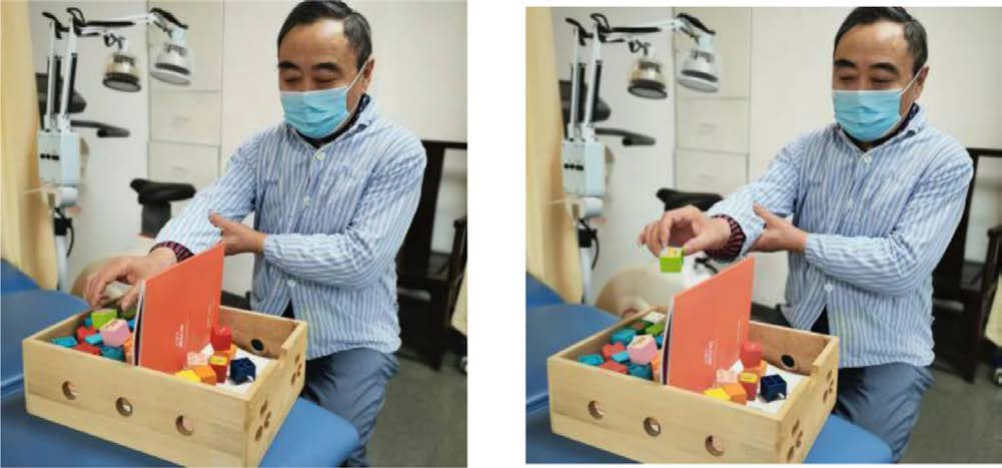
The figure shows the box and block test (BBT) test. The test box contained 150 small wooden cubes of different colors, measuring 25 cm × 25 cm × 25 cm. Effects of Maitland mobilization technique on upper extremity function in stroke survivors with spasticity.

BBT is often used to test the overall dexterity of the hand in stroke patients.^[17]^ The test box contained 150 small wooden cubes of different colors, measuring 25 cm × 25 cm × 25 cm. Subjects were asked to move small squares from one end of the box to the other as quickly as possible. Patients were asked to raise their arms to complete the task and record the number of squares moved in 1 minute (Fig. 2).

#### 2.6.3. Brunnstrom stage

Brunnstrom stage is the theory of “six recovery stages” proposed by Swedish physiotherapist Signe Brunnstrom based on the different stages of motor function recovery in stroke patients,^[18]^ namely, the flaccid stage of hypotonia (stage I), the spastic stage of muscle tension increasing from low to high and gradually appearing combined reaction (stage II); Spasticity gradually became prominent, and synergy movement reached its peak (stage III); with the completion of synergy movement, partial dissociative movement and fine movement appeared (stage IV); dissociative movement continued to develop (stage V); limb recovery was close to normal, and coordination and speed were not good (stage VI).

#### 2.6.4. Modified Ashworth scale

The scale is used to grade the muscle tension and spasm of stroke patients according to the resistance of bare hand test joints during passive movement (divided into 6 grades: 0, 1, 1 +, 2, 3, and 4).^[19]^ MAS grade 0 indicates obvious abnormal muscle tension of limbs, while MAS grade 4 indicates that joints cannot move passively in flexion or extension due to excessive muscle tension.

#### 2.6.5. Functional independence measure

This scale is used to evaluate the independent living ability of stroke patients.^[20]^ It consists of 6 items, 4 for motor ability, and 2 for cognitive ability, divided into 18 detailed motor categories. In addition, based on the patient’s level of motor performance, a score of 7 levels can also be obtained. If the patient needs complete assistance in executing all projects, they will get 18 points; if the patient is able to independently execute all projects, they will get 126 points.

### 2.7. Statistical analysis

All clinical data were processed by SPSS 25.0 statistical software package. For descriptive statistical analysis, the qualitative indicators were described by frequency table and percentage; the quantitative indicators conforming to normal distribution (or approximate normal distribution) were described by mean ± standard deviation (M ± D), and the non-normal distribution data were described by Median (P_25_, P_75_). Comparative analysis within the 2 groups, quantitative data in line with the normal distribution with paired *t* test, not in line with the normal distribution or rank data with Wilcoxon *W* rank sum test. For the comparative analysis between the 2 groups, *χ*^2^ test was used for the qualitative data, and nonparametric test was used for the grade data; *t* test was used for the quantitative data that were in line with the normal distribution (Satterthwaite method was used for the corrected *t* test when the variance was uneven), and Mann–Whitney *U* test rank sum test was used for the quantitative data that were not in line with the normal distribution. The test level was set at 0.05, and *P* < .05 was statistically significant.

## 3. Results

### 3.1. General characteristics

A total of 74 subjects who met the criteria were recruited and voluntarily signed the informed consent. The subjects were randomly divided into the control group and the experimental group in equal proportion. At the end of the 8-week experiment, 71 valid data were collected (1 patient in the control group withdrew from the experiment because of poor blood pressure control, 1 patient in the control group withdrew from the experiment because of medical intervention due to the onset of knee arthritis; 1 patient in the experimental group was excluded because of incomplete statistics), including 35 cases in the control group and 36 cases in the experimental group. There was no significant difference in gender distribution, hemiplegic side (left/right), diagnosis (ischemic stroke/hemorrhagic stroke), past history (smoking history/coronary disease/hypertension/diabetes), age, duration, body mass index (BMI), and mini-mental state examination (MMSE) by statistical analysis (*P* > .05), so the data of the 2 groups were comparable (Table 1).

**Table 1:** Baseline data in patients.

Baseline	Control (n = 35)	Experimental (n = 36)	*P* value
Sex			
Female	25	19	.144
Male	10	17	
Hemiplegic side			
Left	12	7	.189
Right	23	29	
Diagnosis			
Ischemic stroke	13	9	.312
Hemorrhagic stroke	22	27	
Previous history (N/Y)			
Smoking history	30/5	25/11	.155
Coronary disease	26/9	23/13	.443
Diabetes	19/16	23/13	.474
Hypertension	23/12	17/19	.153
Age (yr)	63.20 (7.15)	64.06 (6.03)	.587
Duration (mo)	6.77 (1.94)	6.81 (1.85)	.940
BMI	21.63 (2.23)	21.23 (2.15)	.445
MMSE	28.34 (1.19)	28.64 (1.02)	.263

BMI = body mass index, MMSE = mini-mental state examination.

### 3.2. Changes in upper extremity function

#### 3.2.1. Comparison between 2 groups of patients before the experiment

Before the experiment, there was no statistically significant difference in FMA-UE, BBT, Brunnstrom stage, MAS (shoulder, elbow, wrist, and finger), and FEM scores between the 2 groups of patients (*P* > .05) (Table 2). Therefore, it is feasible to exclude bias in subsequent intervention effect comparisons.

**Table 2:** The results of FMA-UE, BBT, Brunnstrom stage, MAS, and FIM.

Variables	Group	Preintervention	Postintervention	*P* value
FMA-UE Mean (SD)	Control (n = 35)	28.34 (3.105)	32.57 (3.791)	<.001^a^
	Experimental (n = 36)	28.44 (3.376)	34.39 (3.751)	<.001^a^
	*P* value	.895	.046^b^	
BBT Median (P_25_, P_75_)	Control (n = 35)	7 (5,9)	11 (9,13)	<.001^a^
	Experimental (n = 36)	7 (5,8)	13 (12,14)	<.001^a^
	*P* value	.969	.039^b^	
Brunnstrom stage Median (P_25_, P_75_)	Control (n = 35)	3 (2,3)	3 (2,3)	.046^a^
	Experimental (n = 36)	3 (2,3)	3 (3,3)	.025^a^
	*P* value	.316	.186	
MAS-Shoulder Median (P_25_, P_75_)	Control (n = 35)	2 (2,2)	2 (2,2)	.046^a^
	Experimental (n = 36)	2 (1,2)	2 (1,2)	.025^a^
	*P* value	.833	.956	
MAS-Elbow Median (P_25_, P_75_)	Control (n = 35)	2 (2,2)	2 (1,2)	.317
	Experimental (n = 36)	2 (2,2)	2 (2,2)	.157
	*P* value	.652	.773	
MAS-Wrist Median (P_25_, P_75_)	Control (n = 35)	2 (1,2)	2 (1,2)	.083
	Experimental (n = 36)	2 (2,2)	2 (1,2)	.046^a^
	*P* value	.676	.821	
MAS-Finger Median (P_25_, P_75_)	Control (n = 35)	2 (2,2)	2 (2,2)	.157
	Experimental (n = 36)	2 (2,2)	2 (2,2)	.083
	*P* value	.505	.615	
FIM Mean (SD)	Control (n = 35)	59.37 (3.797)	60.58 (4.108)	<.001^a^
	Experimental (n = 36)	65.80 (3.894)	67.89 (3.970)	<.001^a^
	*P* value	.201	.028^b^	

BBT = box and block test, FIM = functional independence measure, FMA-UE = Fugl-Meyer motor assessment-upper extremity, MAS = modified Ashworth scale.

a *P* < .05, intragroup comparison after treatment.

b *P* < .05, intergroup comparison after treatment.

#### 3.2.2. Intergroup comparison after treatment

The FMA-UE scores of the 2 groups of patients after the experiment were 32.57 (3.791) and 34.39 (3.751), respectively. The BBT scores were 11 (9,13) and 13 (12,14), and the FIM scores were 60.58 (4.108) and 67.89 (3.970), with significant differences (*P* values of .046, .039, and .028, respectively). However, there was no significant difference in Brunnstrom stage and MAS between the 2 groups (*P* > .05) (Table 2).

#### 3.2.3. Intragroup comparison after treatment

There were significant differences in FMA-UE, BBT, and FIM between the 2 groups of patients after the experiment compared to before (*P* < .001), while the improvement in the experimental group was more significant than that in the control group; there were significant differences in Brunnstrom Stage and MAS-Shoulder between the 2 groups of patients after the experiment and before the experiment (*P* < .05); However, there was no statistically significant difference (*P* > .05) between the 2 groups of patients in terms of MAS-Elbow, MAS-Wrist, and MAS-Finger after the experiment compared to before (Table 2).

## 4. Discussion

Spasticity is a motor dysfunction caused by excessive excitation of stretch reflex, which is characterized by speed-dependency. It is a common complication of central nervous system injury and one of the important factors leading to disability in stroke patients. At present, Bobath, PNF and other neurodevelopmental therapies and active exercise training are the main rehabilitation therapies for spasticity in clinic practice. The abnormal muscle tone of the hemiplegic upper extremity of stroke patients can destroy the balance of bone, joint, and soft tissue of the limb for a long time, which can lead to the “tendon off-position, joint subluxation” model advocated by the field of orthopedics and traumatology in traditional Chinese medicine. The treatment of limb joint spasm after stroke should be combined with the passive mobilization treatment of bone and joint structure on the basis of neurodevelopmental therapy with soft tissue intervention as the main treatment, which can achieve the effect of correcting the imbalance of upper extremity muscles and bones on the hemiplegic side.

In Table 2, the FMA-UE scores, BBT scores and FIM scores of the 2 groups of patients after the experiment had significant differences (*P* values of .046, .039, and .028, respectively). It indicates that 8 weeks of conventional rehabilitation treatment combined with Maitland joint mobilization can effectively improve the FMA-UE, BBT, and FIM scores of stroke patients, and compared with the control group, it is more significant in improving the upper extremity function of the hemiplegic side and improving the ability of daily living. Yet there was no significant difference in Brunnstrom stage and MAS between the 2 groups (*P* > .05) (Table 2).

Maitland joint mobilization technique is a conventional method in the field of modern musculoskeletal manual therapy, which can effectively improve the pain of joint and limited movement. This technique is a passive manipulation performed by the therapist within the allowable range of joint movement, which can be divided into 4 grades, of which grade I and II are used for pain-related limitation of movement, grade III is used for joint pain accompanied by stiffness, and grade IV is used to improve the limitation of movement caused by soft tissue adhesion contracture. For stroke patients with upper extremity spasticity, the joints of spastic extremities are often stiff and painful, which also hinders the conduction of normal proprioception and the recovery of normal movement patterns. Maitland joint mobilization improves the range of motion of the joint while manipulating the stiff joint, and squeezing the joint can help to improve the proprioception of the extermity,^[21]^ which is similar to the spiral diagonal pattern unique to PNF technology, and the combination of the 2 treatments has a superimposed effect. Neurodevelopmental therapy mainly focuses on the stimulation of soft tissue and the inhibition of abnormal nerve reflexes. Maitland joint mobilization technique can make up for the deficiency of neurodevelopmental therapy in bone and joint manipulation to a certain extent, and also promote the reconstruction of normal limb movement patterns.^[22]^ It is hypothesized that the Maitland joint mobilization also modulates limb spasticity after stroke by a variety of mechanisms, such as modulating mutual inhibition, decreasing excitability of the stretch reflex, and increasing presynaptic inhibition, thereby enhancing plasticity in the injured brain.

There was no significant difference in MAS between the 2 groups (*P* > .05) (Table 2). There was a significant difference in MAS-Shoulder between the 2 groups after the experiment (*P* < .05), while there was no significant difference in MAS-Elbow, MAS-Wrist, and MAS-Finger between the 2 groups before the experiment (*P* > .05). This may be related to the neurophysiological principle of upper extremity function recovery in stroke patients. On the one hand, because the repair of brain injury is a complex and slow process, the improvement of spasm is a difficult point in the field of clinical neurological rehabilitation.^[23]^ On the other hand, the normal motor nerve development sequence of human body follows the principle from proximal end to distal end,^[24,25]^ which is consistent with the recovery principle of limb joints after stroke. Therefore, the improvement of shoulder joint function at the proximal end of the limb is theoretically earlier than the recovery of elbow joint and hand function.^[26]^ In addition, it may also be related to the limitations of this study. In this study, only 8 weeks of intervention were carried out, and more long-term intervention and long-term follow-up can provide higher quality evidence. Therefore, we should increase the exploration in this area in the future.

At the same time, there are some limitations in this study that need to be noted. Although the observation indexes adopted are the most widely used scales in clinical practice, most of them are qualitative and semiquantitative assessments, which inevitably lead to measurement bias. In order to solve this problem, in the future, the application potential of joint mobilization technology in improving upper extremity function in stroke patients can be demonstrated more objectively from multiple perspectives by means of isokinetic muscle strength testing system^[27,28]^ and quantitative evaluation methods such as neuroelectrophysiology (H reflex, *F* wave,^[29,30]^ and surface electromyography^[31]^).

## 5. Conclusions

In this study, the Maitland joint mobilization technique was applied to the conventional rehabilitation treatment of stroke patients with upper extremity spasticity, and the results showed that the passive mobilization treatment of spasticity joint had a significant improvement in upper extremity function compared with no intervention. The results can be used as a reference for physical therapists to formulate more perfect neurological rehabilitation treatment plans in the future.

## Author contributions

**Conceptualization:** Ziqing Liu.

**Formal analysis:** Ziqing Liu, Zhangjie Li, Chaoyang Duan.

**Investigation:** Ziqing Liu, Zhangjie Li, Chaoyang Duan.

**Methodology:** Ziqing Liu.

**Project administration:** Ziqing Liu, Chaoyang Duan.

**Supervision:** Ziqing Liu, Chaoyang Duan.

**Writing – original draft:** Ziqing Liu, Zhangjie Li, Chaoyang Duan.

**Writing – review & editing:** Ziqing Liu, Chaoyang Duan.

**Data curation:** Zhangjie Li, Chaoyang Duan.
